# Efficacy and safety of combined prolonged-release oxycodone and naloxone in the management of moderate/severe chronic non-malignant pain: results of a prospectively designed pooled analysis of two randomised, double-blind clinical trials

**DOI:** 10.1186/1472-6904-10-12

**Published:** 2010-09-29

**Authors:** Oliver Löwenstein, Petra Leyendecker, Eberhard A Lux, Mark Blagden, Karen H Simpson, Michael Hopp, Björn Bosse, Karen Reimer

**Affiliations:** 1 Schmerzpraxis, Rheinstrasse, Mainz, Germany; 2 Mundipharma Research GmbH & Co. KG, Hoehenstrasse, Limburg (Lahn), Germany; 3 Klinik für Schmerz- und Palliativmedizin, Klinikum St. Marien-Hospital Lünen, Altstadtstrasse, Lünen, Germany; 4 Avondale Surgery, Chesterfield, Avondale Road, Derbyshire, UK; 5 Leeds Teaching Hospital, Glebe House, Scholes Lane, Leeds, UK; 6 University Witten/Herdecke, Alfred-Herrhausen-Straße, Witten, Germany

## Abstract

**Background:**

Two randomised 12-week, double-blind, parallel-group, multicenter studies comparing oxycodone PR/naloxone PR and oxycodone PR alone on symptoms of opioid-induced bowel dysfunction in patients with moderate/severe non-malignant pain have been conducted.

**Methods:**

These studies were prospectively designed to be pooled and the primary outcome measure of the pooled data analysis was to demonstrate non-inferiority in 12-week analgesic efficacy of oxycodone PR/naloxone PR versus oxycodone PR alone. Patients with opioid-induced constipation were switched to oxycodone PR and then randomised to fixed doses of oxycodone PR/naloxone PR (n = 292) or oxycodone PR (n = 295) for 12 weeks (20-80 mg/day).

**Results:**

No statistically significant differences in analgesic efficacy were observed for the two treatments (p = 0.3197; non-inferiority p < 0.0001; 95% CI -0.07, 0.23) and there was no statistically significant difference in frequency of analgesic rescue medication use. Improvements in Bowel Function Index score were observed for oxycodone PR/naloxone PR by Week 1 and at every subsequent time point (-15.1; p < 0.0001; 95% CI -17.3, -13.0). AE incidence was similar for both groups (61.0% and 57.3% of patients with oxycodone PR/naloxone PR and oxycodone PR alone, respectively).

**Conclusions:**

Results of this pooled analysis confirm that oxycodone PR/naloxone PR provides effective analgesia and suggest that oxycodone PR/naloxone PR improves bowel function without compromising analgesic efficacy.

**Trial registration numbers:**

ClinicalTrials.gov identifier: NCT00412100 and NCT00412152

## Background

Opioids are established treatment for moderate/severe chronic malignant pain, as recommended by the World Health Organization (WHO) [1]; furthermore, they are the mainstay of treatment for chronic non-malignant pain [2]. Oxycodone is a semi-synthetic, opioid that is effective in alleviating malignant pain [3,4], postoperative pain, osteoarthritis [5] and neuropathic non-malignant pain [6-8].

Opioids exert their analgesic effects mainly by binding to receptors within the central nervous system; however, opioid receptors also reside within the gastrointestinal (GI) tract [9]. Binding of opioids to these receptors commonly leads to GI adverse events (AEs), including straining, incomplete evacuation, bloating, abdominal distension and gastric reflux that are collectively known as opioid-induced bowel dysfunction[10]. Opioid-induced constipation (OIC) is the most frequently reported AE in patients receiving opioids [2]. Unlike most side effects associated with opioids that subside with chronic use, constipation often persists and usually requires active management [11]. The pain and discomfort caused by OIC can cause patients to reduce or discontinue their opioids [9], resulting in inadequate analgesia and poor quality of life; up to 30% of patients reduce or discontinue opioids due to such problems [12].

Current management for OIC is nonspecific and often ineffective [9]. Laxatives can improve symptoms in some patients, although many do not achieve adequate relief of symptoms [9,10]. In one survey, only 46% of patients taking medication for OIC experienced improvement for over 50% of the time compared with 80% of non-opioid users taking similar medication for constipation [10]. This may be because laxatives do not counteract the underlying opioid receptor-mediated mechanism of OIC. Prevention of OIC, and bowel dysfunction, is therefore a more effective strategy than treating it once it occurs [9].

An emerging strategy for targeting the cause of OIC is oral administration of opioid-receptor antagonists that act specifically and locally within the GI tract. These prevent or minimize adverse GI effects, but do not compromise central analgesic opioid effects because of their limited systemic bioavailability. Naloxone is an opioid-receptor antagonist that, when administered orally, has a very low systemic bioavailability (< 3%) due to its extensive first-pass hepatic metabolism, but acts on opioid receptors within the GI tract [13].

Immediate-release (IR) oral naloxone was evaluated in several studies to evaluate its ability to reduce OIC [14-17]. However, results have been equivocal and in some of these study results showed, that even low doses of oral IR naloxone were causing withdrawal symptoms. A prolonged-release (PR) formulation of oral naloxone can reveal a reduction of these risks. The extensive clinical development with the PR formulation of the fixed-dose combination oxycodone (PR)/naloxone (PR) confirmed the favourable efficacy and safety of this combination also with respect to the withdrawal and analgesia [18-23].

Results from a study which compared pharmacokinetics data from a single-dose and multiple-dose bioequivalence study of fixed-dose combination (FDC) oxycodone prolonged-release (PR)/naloxone PR versus separate formulations of oxycodone PR and naloxone PR administered concurrently in healthy volunteers, demonstrated that the co-administration of oxycodone PR and naloxone PR in a FDC does not significantly affect the bioavailability of either of its constituents [20]. Indeed, oral co-administration of oxycodone PR/naloxone PR in a 2:1 ratio provides effective analgesia for patients with severe chronic pain while significantly improving OIC [21]. A previous Phase III trial showed that the fixed combination of oxycodone PR/naloxone PR was superior to placebo in analgesic efficacy and provided benefits with regard to bowel function. Analgesic efficacy of oxycodone PR/naloxone PR was comparable to that of oxycodone PR, which was included as an active comparator. Therefore, the addition of naloxone PR to oxycodone PR in the fixed combination did not negatively impact the analgesic efficacy of the oxycodone component [19]. In addition, results from a prospective non-interventional, 4-week observational study which was designed to evaluate the efficacy and safety of oxycodone PR/naloxone PR reported that this combination proved effective and safe in more than 7000 patients with severe pain of different aetiologies, including those with severe cancer pain [24]. These findings have been further corroborated in elderly patient subgroups and several long-term extension phases of clinical trials [18,25,26]. Moreover, naloxone PR/oxycodone PR has been shown to improve patient assessment of analgesic opioid therapy for severe chronic pain, in terms of both efficacy and tolerability [23].

Here, we present a prospectively planned pooled analysis of data from two Phase III studies that included subjects with OIC [27,28]. Both studies were similar in design and were conducted to compare the safety and efficacy of the fixed combination of oxycodone PR/naloxone PR (oxycodone PR 20-80 mg/day) compared with oxycodone PR in the treatment of patients with moderate-to-severe chronic non-malignant pain. The primary focus of the pooled analysis was to examine the analgesic efficacy of oxycodone PR/naloxone PR compared with oxycodone PR alone during 12 weeks of treatment.

## Methods

This was a prospectively designed pooled analysis of two randomised, double-blind, double-dummy, parallel-group, multicenter, 12-week studies (OXN3001 and OXN3006). Details of the designs of the two studies have been published previously [27,28]. The primary endpoint of the pooled data analysis was to demonstrate the non-inferiority of oxycodone PR/naloxone PR compared with oxycodone PR alone in terms of 12-week analgesic efficacy in patients with moderate/severe non-malignant pain; this was based on the patients' average pain over the previous 24 hours, assessed at each double-blind study visit using the Pain Intensity Scale.

Patients received fixed doses of study medication containing oxycodone PR doses of 20-80 mg/day. Secondary objectives were to determine the frequency of rescue medication used per day and to investigate the symptoms of constipation based on laxative intake and patient bowel function self-assessment using the validated Bowel Function Index (BFI) [29]. Another secondary objective was to assess the safety of oxycodone PR/naloxone PR and oxycodone PR. Exploratory analyses included an assessment of overall health of the treatment groups, using the Short-Form (SF)-36version 2 questionnaire [30,31] and the Treatment Satisfaction Questionnaire (TSQM) [32].

The pooled analysis of the two studies was prospectively planned to obtain a sample size providing adequate statistical power to confirm analgesic non-inferiority of oxycodone PR/naloxone PR and oxycodone PR alone (primary endpoint). Furthermore, analysis of the pooled data allows the exploration of the efficacy and safety of the entire dose range of oxycodone PR/naloxone PR compared with oxycodone PR. The studies were conducted in accordance with the Declaration of Helsinki (1964) and all of its accepted amendments to date [33] as well as complying with the principles of Good Clinical Practice set by the International Conference on Harmonization [34] and the European Union Clinical Trials Directive (2001) [35]. Written informed consent was obtained from all participants at screening.

### Patient population

Males and females aged ≥ 18 years were recruited into the two studies if they had a documented history of moderate/severe non-malignant pain that required continual opioid therapy (oxycodone equivalent of ≥ 20 mg/day and ≤ 80 mg/day), had constipation that was caused or aggravated by an opioid and were likely to benefit from WHO step III opioid therapy for the duration of the study [1].

The subject's subjective assessment of their opioid-induced constipation had to be confirmed at screening by interviews performed by the investigator. In cases where there was a close relationship between the opioid intake and the occurrence/aggravation of constipation, the existing constipation was regarded as caused or aggravated by opioids. Furthermore a definition describing constipation caused or aggravated by opioids has been developed based on the Rome II criteria [36] and these criteria had to be fulfilled prior to randomisation.

Exclusion criteria included history of hypersensitivity to oxycodone, naloxone, related products or other substances; any contraindication to bisacodyl or other substances in the study laxative; females who were pregnant or lactating; patients with malignancy-related pain, rheumatoid arthritis or evidence of clinically unstable disease or of impaired liver and/or kidney function at study entry; evidence of significant structural abnormalities of the GI tract; or any diseases/conditions that affected bowel transit.

### Study design

**Figure 1:**
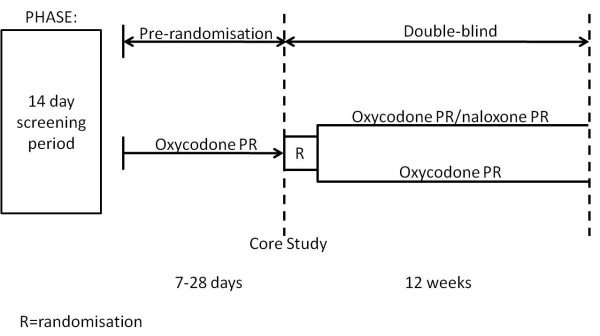
**Study design**. PR = prolonged-release.

The studies consisted of a screening phase, and a pre-randomisation phase followed by a double-blind phase in which patients received the study medication for up to 12 weeks (Figure 1). During the pre-randomisation run-in phase, patients were switched to oxycodone PR alone over 7 to 28 days, during which they were titrated to an effective analgesic dose. Patients were also switched to the standard laxative regimen using oral bisacodyl and were permitted to use oxycodone immediate-release during the run-in phase.

At study baseline (Visit 3), all patients had achieved stable pain control, had confirmed OIC and had satisfied all other inclusion and exclusion criteria. Patients were then randomised to double-blind study medication: oxycodone PR/naloxone PR (2:1 fixed-dose ratio) or oxycodone PR alone. Patients who received more than two doses of rescue medication per day for persistent pain were permitted to increase their dose of study medication (oxycodone PR component) up to 120 mg/day during the double-blind phase. Since the studies were double-dummy, oxycodone PR placebo was given to patients in the combination treatment group, and oxycodone PR/naloxone PR placebo was given to those in the oxycodone PR alone treatment group.

### Efficacy assessments

Efficacy data were collected in daily diaries and during eight site visits. The primary efficacy variable was the Pain Intensity Scale [37] that assesses patients' pain on an 11-point ordinal scale (0 = no pain, 10 = pain as bad as you can imagine). Secondary endpoints included frequency of rescue medication administration (oxycodone immediate-release [IR], prescribed every 4 hours as needed), patient diary assessment of laxatives (bisacodyl) taken in the first 4 weeks of the double-blind phase and a patient bowel function self-assessment using the validated BFI [29]. The BFI is rated as the mean score on the following items: ease of defecation (0-100; 0 = easy/no difficulty, 100 = severe difficulty), feeling of incomplete bowel evacuation (0-100; 0 = not at all, 100 = very strong), and judgment of constipation (0-100; 0 = not at all, 100 = very strong). Exploratory endpoints included the SF-36 health survey and the TSQM, which assess general health and patient satisfaction, respectively.

### Safety assessments

Safety assessments consisted of monitoring and recording all AEs and serious AEs (SAEs), and monitoring haematology, blood chemistry, urine values and vital signs, and performing physical examinations.

### Statistical analysis

The full analysis population consisted of those patients who were randomised and received at least one dose of study medication during the double-blind phase and who had at least one double-blind assessment of the primary efficacy variable. The per-protocol population included those patients without any major protocol deviations, while the safety population included all randomised patients who received any study medication and who had at least one post-baseline safety assessment.

The primary efficacy variable was analyzed within a mixed model repeated measurements (MMRM) analysis. The MMRM analysis included terms for study, treatment, time as categorical variables, and pre-randomisation pain at the end of the titration period and subject as random effects. The primary comparison was the non-inferiority analysis between oxycodone PR/naloxone PR and oxycodone PR, at a one-sided 2.5% level of significance. Two-sided 95% confidence intervals (CIs) between oxycodone PR/naloxone PR and oxycodone PR were calculated for the per-protocol and full analysis populations. The primary comparison was based on the per-protocol population and non-last observation carried forward (non-LOCF; i.e. observed cases) and confirmed using the full analysis population with LOCF and non-LOCF.

Secondary efficacy variables included the BFI, which was analyzed via the same methods as the primary endpoint. Descriptive statistics were used to describe the frequency of rescue medication intake and the number of patients taking laxatives during the entire or first 4 weeks of the double-blind phase. The treatment groups were compared using Fisher's exact test for the number of patients taking laxatives during the first 4 weeks of the double-blind phase.

The patient incidence (%) and number of reports of treatment-emergent AEs were calculated and presented for each treatment using MedDRA preferred term and body system. Absolute values and changes to baseline of vital signs and laboratory parameters were analyzed using descriptive statistics.

## Results

**Figure 2:**
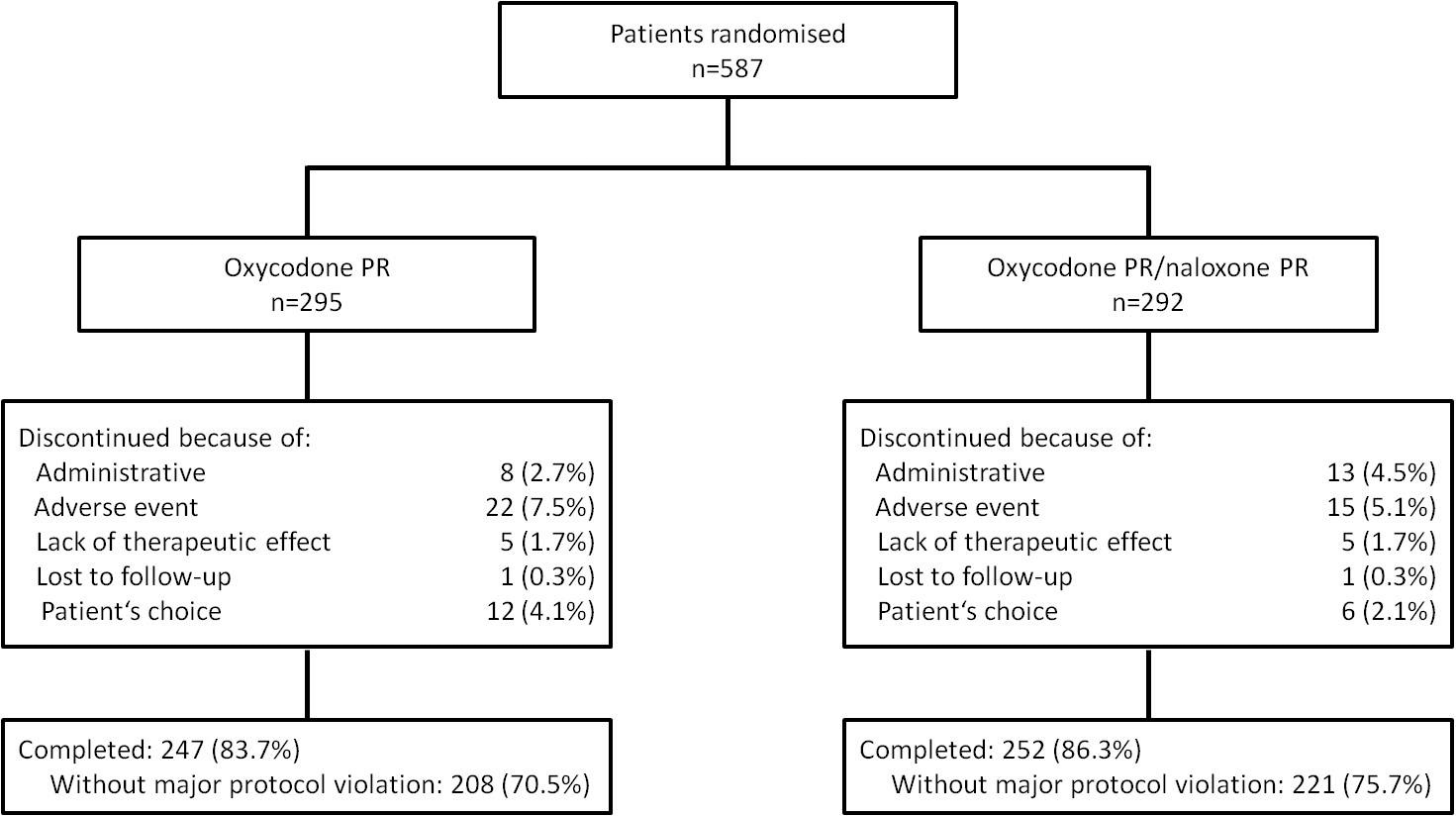
**Patient disposition**. PR = prolonged-release

859 patients were enrolled in the two studies, of these 587 patients were randomised to treatment in the double-blind phase and were included in the safety analysis; 581 patients received at least one dose of study medication and were included in the full analysis population. 499 patients (85%) completed the studies, of which 429 qualified for the per-protocol population (Figure 2). Similar rates of study drug discontinuation were observed in the two treatment groups: 16.3 and 13.7%, respectively, in the oxycodone PR/naloxone PR and the oxycodone PR groups. The main reason for early discontinuation was AEs (Figure 2).

All patients reported moderate/severe chronic non-malignant pain requiring continuous opioid therapy, with constipation caused or aggravated by an opioid. The majority of patients was pre-treated with WHO III opioids and received individual laxative regimen. After the switch to the standard laxative regimen (oral biscacodyl), doses of bisacodyl were 10 mg per intake. However, investigators instructed their subjects that, if they experienced discomfort during the 72 hour period, they could take bisacodyl as a laxative within 72 hours of their most recent bowel movement, as required, to treat constipation. At the discretion of the Investigator, the bisacodyl dose could be lowered (to 5 mg) if either the investigator or the subject felt that dose was sufficient to provide adequate bowel movement.

The majority of patients (86%) presented with pain associated with musculoskeletal and connective tissue disorders; the most frequent conditions were back pain, osteoarthritis and intervertebral disc disorders. Many patients (34%) reported neuropathic pain e.g. neuralgia, sciatica and cervicobrachial syndrome and the presence of more than one type of chronic pain was possible. There were no differences in patient demographics between the two treatment groups; although there were more females than males in the studies, the ratio of women to men was similar in the two patient groups (Table 1).

**Table 1:** Patient demographics at baseline (Safety population)

		Oxycodone PR (n = 295)	Oxycodone PR/naloxone PR (n = 292)	Total (n = 587)
Age (years)	Mean (SD)	58.3 (11.52)	57.5 (11.27)	57.9 (11.40)
	Median	58	58	58
	Min, Max	25, 87	29, 84	25, 87

Age group, n (%)	≤ 65	216 (73.2)	221 (75.7)	437 (74.4)
	> 65	79 (26.8)	71 (24.3)	150 (25.6)

Sex, n (%)	Male	108 (36.6)	102 (34.9)	210 (35.8)
	Female	187 (63.4)	190 (65.1)	377 (64.2)

Race, n (%)	Caucasian	293 (99.3)	292 (100.0)	585 (99.7)
	Black	1 (0.3)	0 (0.0)	1 (0.2)
	Other	1 (0.3)	0 (0.0)	1 (0.2)

Weight (kg)	Mean (SD)	84.7 (20.52)	84.2 (18.03)	84.4 (19.31)
	Median	83	80.6	82
	Min, Max	44, 174	47.5, 147	44, 174

PR = prolonged-release; SD = standard deviation

### Efficacy evaluation

The primary objective was to demonstrate the non-inferiority of oxycodone PR/naloxone PR to oxycodone PR alone for analgesic efficacy (average pain over the previous 24 hours), as assessed using the Pain Intensity Scale. Efficacy analysis was based on the per-protocol analysis (non-LOCF). Over the course of the 12-week, double-blind phase (Visit 3 to Visit 8) mean Pain Intensity Scale scores remained stable, with no statistically significant difference in analgesic efficacy observed between the two treatment groups (p = 0.3197; non-inferiority p < 0.0001; 95% CI -0.07, 0.23; Table 2). This indicated that oxycodone PR/naloxone PR was non-inferior to oxycodone PR. Non-inferiority for the mean Pain Intensity Scale scores was confirmed in the full analysis population (p = 0.9042; non-inferiority p < 0.0001; 95% CI -0.14, 0.13; LOCF) and (p = 0.8779; non-inferiority p < 0.0001; 95% CI -0.15, 0.13; non-LOCF). Furthermore, the majority of patients (77%) continued on the same dose of study medication from the time of randomisation to the end of the double-blind phase; in each treatment group, 65 patients were up-titrated and three were down-titrated. The mean (SD) daily supplemental analgesic use was 0.5 (0.63) uses in the oxycodone PR group and 0.6 (0.65) in the oxycodone PR/naloxone PR group between Days 1-28. During Days 57-84, the mean (SD) supplemental analgesic use was 0.4 (0.61) in the oxycodone PR group and 0.4 (0.58) in the oxycodone PR/naloxone PR group. There was no statistically significant difference in mean daily use of supplemental rescue analgesic medication between those treated with oxycodone PR/naloxone PR or those who received oxycodone PR for the both the per-protocol (treatment difference: -0.05; p = 0.39; 95% CI -0.15, 0.06) and full analysis populations (treatment difference:-0.05; p = 0.3386; 95% CI -0.14, 0.05).

**Table 2:** Mean pain intensity over the previous 24 hours at each study visit (per-protocol population; non-last observation carried forward analysis)

Name (visit)	Pain Intensity Scale score			
		Oxycodone PR	Oxycodone/Naloxone PR	Total
**Randomisation** **(Visit 3)**	N	208	221	429
	Mean (SD)	3.3 (0.97)	3.4 (1.07)	3.4 (1.02)
	Median	3.0	3.0	3.0
	Min, Max	0.0, 7.0	0.0, 9.0	0.0, 9.0

**Week 1** **(Visit 4)**	N	208	220	428
	Mean (SD)	3.5 (1.3)	3.6 (1.51)	3.6 (1.41)
	Median	3.0	4.0	4.0
	Min, Max	0.0, 7.0	0.0, 9.0	0.0, 9.0

**Week 4** **(Visit 6)**	N	208	220	428
	Mean (SD)	3.5 (1.37)	3.5 (1.51)	3.5 (1.44)
	Median	3.0	4.0	3.5
	Min, Max	0.0, 7.0	0.0, 9.0	0.0, 9.0

**Week 12** **(Visit 8)**	N	204	220	424
	Mean (SD)	3.5 (1.53)	3.6 (1.78)	3.6 (1.67)
	Median	4.0	4.0	4.0
	Min, Max	0.0, 8.0	0.0, 9.0	0.0, 9.0

Min = minimum; Max = maximum; PR = prolonged-release; SD = standard deviation

**Figure 3:**
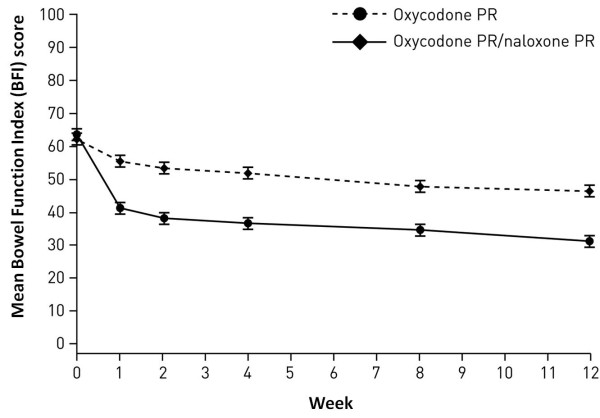
**Mean Bowel Function Index score over time (full analysis population; last observation carried forward analysis)**.

A secondary objective of the study was to assess symptoms of constipation. At baseline (Visit 3), bowel function, assessed using the BFI, was comparable between the two treatment groups (Figure 3). Subsequently, statistically significant and clinically relevant improvements [29] in the oxycodone PR/naloxone PR group were observed by Week 1 (Visit 4) and at every subsequent time point during the 12-week, double-blind study period (-15.1; p < 0.0001; CI -17.3, -13.0; Figure 3).

Occurrence of constipation was also assessed by considering the number of patients taking laxatives. Patients treated with oxycodone PR/naloxone PR had a significantly lower laxative intake than patients who received oxycodone PR alone (p < 0.0001) and, during the first 4 weeks of the double-blind period, significantly fewer patients treated with oxycodone PR/naloxone PR required laxatives compared to the oxycodone PR group (36.5 vs 59.0%, respectively; p < 0.0001).

There were no notable differences in SF-36 scores between the two treatment groups, including the SF-36-Bodily Pain subscale, at Visits 2 and 8 (Week 12). During the 12-week, double-blind period, there were no significant differences in TSQM scores for those patients who received oxycodone PR/naloxone PR or oxycodone PR.

### Safety

Overall, the incidence of AEs was similar between the groups (Table 3), with a total of 61.0 and 57.3% of patients experiencing AEs with oxycodone PR/naloxone PR and oxycodone PR alone, respectively. The number of patients experiencing AEs related to study medication was similar between the two groups (31.9 and 36.0% with oxycodone PR/naloxone PR and oxycodone PR alone, respectively). A similar number of patients discontinued because of AEs in the oxycodone PR and oxycodone/naloxone PR groups (6.4 and 4.8% respectively), and there were no deaths during the study.

**Table 3:** Incidence of adverse events by organ class (≥ 10%) and preferred term (≥ 1%; Safety population)

	Oxycodone PR n = 295 n (%)	Oxycodone/naloxone PR n = 292 n (%)	Total n = 587 n (%)
**Gastrointestinal disorders**	**64 (21.7)**	**61 (20.9)**	**125 (21.3)**

Dry mouth	3 (1.0)	3 (1.0)	6 (1.0)

Diarrhoea	11 (3.7)	15 (5.1)	(26) 4.4

Constipation	10 (3.4)	2 (0.7)	12 (2.0)

Upper abdominal pain	4 (1.4)	6 (2.1)	10 (1.7)

Abdominal pain	7 (2.4)	11 (3.8)	18 (3.1)

Vomiting	8 (2.7)	5 (1.7)	13 (2.2)

Nausea	25 (8.5)	23 (7.9)	48 (8.2)

**General disorders and administrative site conditions**	**26 (8.8)**	**34 (11.6)**	**60 (10.2)**

Pain	5 (1.7)	9 (3.1)	14 (2.4)

Peripheral oedema	4 (1.4)	5 (1.7)	9 (1.5)

Fatigue	7 (2.4)	6 (2.1)	13 (2.2)

Chills	3 (1.0)	5 (1.7)	8 (1.4)

**Infections and infestations**	**56 (19.0)**	**47 (16.1)**	**103 (17.5)**

Nasopharyngitis	10 (3.4)	4 (1.7)	103 (17.5)

Lower respiratory tract infection	3 (1.0)	3 (1.0)	6 (1.0)

Gastroenteritis	7 (2.4)	5 (1.7)	12 (2.0)

Bronchitis	3 (1.0)	3 (1.0)	6 (1.0)

Urinary tract infection	6 (2.0)	13 (4.5)	19 (3.2)

**Musculoskeletal and connective tissue disorders**	**34 (11.5)**	**39 (13.4)**	**73 (12.4)**

Back pain	9 (3.1)	11 (3.8)	20 (3.4)

Arthralgia	5 (1.7)	6 (2.1)	11 (1.9)

Myalgia	2 (0.7)	4 (1.4)	6 (1.0)

**Nervous system disorders**	**35 (11.9)**	**40 (13.7)**	**75 (12.8)**

Sciatica	1 (0.3)	6 (2.1)	7 (1.2)

Headache	11 (3.7)	12 (4.1)	23 (3.9)

Dizziness	10 (3.4)	5 (1.7)	15 (2.6)

PR = prolonged-release

The most common AEs were GI (21.3% in the total group). Infections and infestations (17.5%), nervous system disorders (12.8%), musculoskeletal and connective tissue disorders (12.4%), and general disorders (10.2%) were the other most common AEs. Nausea, diarrhoea and abdominal pain were the most frequently reported GI AEs across both groups (Table 3). Constipation (only worsening of existing condition observed prior to randomisation) was reported in more patients treated with oxycodone PR (3.4%) than in those who received oxycodone PR/naloxone PR (0.7%). Overall, there was a low incidence of diarrhoea (4.4% in the total group), with comparable numbers of patients experiencing this in both treatment groups (Table 3). Cases of diarrhoea were generally transient (mean duration 5.81 days).

The incidence of severe AEs (9.9 and 10.8% in the oxycodone PR/naloxone PR and oxycodone PR groups, respectively) and SAEs (4.5 and 4.4%, in the oxycodone PR/naloxone PR and oxycodone PR groups, respectively) was low with both treatments. However, there was a slightly higher incidence of study drug-related SAEs in the oxycodone PR/naloxone PR group than in the oxycodone PR alone group (2.4 and 1.4%, respectively). Three patients (1.0%) in the oxycodone PR group had AEs related to opioid withdrawal, however this was observed in only one patient (0.3%) in the oxycodone PR/naloxone PR group.

Analyses of the mean change in haematology and blood chemistry revealed no changes of clinical concern. Vital signs, including systolic and diastolic blood pressure and pulse rate, were within the normal range at screening and end of study in both treatment groups. There was no change in body weight in either treatment group during the 12-week study.

## Discussion

The results of this prospectively designed pooled analysis demonstrate that during a 12-week period, in this population, oxycodone PR/naloxone PR (20-120 mg/day) provides analgesia that is as effective as oxycodone PR. This was indicated by the non-inferiority of oxycodone PR/naloxone PR versus oxycodone PR in mean Pain Intensity Scale and a low and comparable use of supplemental analgesic medication in both treatment groups. Furthermore, patients receiving oxycodone PR/naloxone PR demonstrated statistically and clinically significant improvements in bowel function. Oxycodone PR/naloxone PR was superior to oxycodone PR with regard to bowel function, particularly in reducing constipation. There was also a significantly reduced use of laxatives in the first 4 weeks of the study in patients who received oxycodone PR/naloxone PR compared with those who received oxycodone PR alone.

The incidence of AEs was comparable in both treatment groups; a similar number of patients in each group discontinued the study because of AEs. The most frequently reported AEs were GI. However, importantly, the incidence of diarrhoea was generally low, transient and comparable between treatment groups. After the administration of oxycodone PR/naloxone PR there were no additional or unexpected risks observed when compared with oxycodone PR treatment. Consequently, the risk/benefit ratio was more favourable for oxycodone PR/naloxone PR compared with oxycodone PR alone in this group of patients.

Two exploratory endpoints (SF-36 and TSQM) showed that there were no differences in quality of life scores between the two groups. These questionnaires measure general health and treatment satisfaction and do not specifically assess pain-related quality of life. However, it can be assumed that patients' responses to these assessments are strongly influenced by pain. Therefore, the comparable scores indicate a similar analgesic efficacy for oxycodone PR/naloxone PR.

One of the main limitations of this analysis was that clinical efficacy and tolerability can only be discussed for the dose ranges used. Further studies are required to establish the clinical benefits of other doses and to assess longer-term effects of oxycodone PR/naloxone PR combination treatment for patients with chronic pain.

## Conclusions

This pooled analysis demonstrated the non-inferiority of oxycodone PR/naloxone PR compared with oxycodone PR alone for analgesic efficacy in patients with moderate-to-severe non-malignant pain treated for 12 weeks. The oxycodone PR/naloxone PR formulation improved bowel function and significantly reduced constipation in these patients.

## List of abbreviations

AE: adverse event; BFI: Bowel Function Index; CIs: confidence intervals; FDC: fixed-dose combination; GI: gastrointestinal; IR: immediate release; Non-LOCF: non-last observation carried forward; OIC: Opioid-induced constipation; PR: prolonged release; SAE: serious adverse event; TSQM: Treatment Satisfaction Questionnaire for Medication; WHO: World Health Organisation

## Competing interests

The authors declare that they have no competing interests.

## Authors' contributions

This study was funded by Mundipharma Research GmbH & Co. KG.

PL, MH, BB and KR are employed by Mundipharma Research GmbH & Co. KG at the time of the study conduct and article drafting. OL and MB were involved as investigators in the clinical study reported here. KHS has received honoraria from medical device and pharmaceutical companies for lectures at conferences, to attend advisory boards, to contribute to publications and to support professional development, e.g. attendance at meetings. KHS is a member of the Leeds Pallium Research Group that has received research funding from medical device and pharmaceutical companies for clinical research. EAL has received honoraria for lectures from Mundipharma, Gruenenthal, Wyeth and Pfizer. All authors were involved in the development, critical reviewing and approval of this manuscript.

## Pre-publication history

The pre-publication history for this paper can be accessed here:

http://www.biomedcentral.com/1472-6904/10/12/prepub
